# Super-recognizers sample visual information of superior computational value for facial recognition

**DOI:** 10.1098/rspb.2025.2005

**Published:** 2025-11-05

**Authors:** James D. Dunn, Victor Varela, Bojana Popovic, Stephanie Summersby, Sebastien Miellet, David White

**Affiliations:** ^1^ School of Psychology, University of New South Wales, Sydney, New South Wales 2052, Australia; ^2^ School of Psychology, University of Wollongong, Wollongong, New South Wales 2522, Australia

**Keywords:** face recognition, individual differences, eye movements, deep neural networks

## Abstract

Super-recognizers—individuals with exceptionally high face recognition abilities—are a key exemplar of biological visual expertise. Recent eye-tracking evidence suggests that their expertise may be driven by exploratory viewing behaviour during learning, but it remains unclear whether this perceptual sampling is functional for face identity processing. Here, we develop a novel approach to quantify the computational value of face information samples and test the utility of information sampling in super-recognizers. Using measurements of eye gaze behaviour, we reconstructed the retinal information that participants acquired while learning new faces. We then evaluated the computational value of this information for face identity processing using nine deep neural networks (DNNs) optimized for this task. Identity matching accuracy improved across all DNNs when using visual information sampled by super-recognizers compared with typical viewers. Interestingly, this advantage could not be explained by the greater quantity of information alone, and so differences in both the quantity and quality of face information encoded on the retina contribute to individual differences in face processing ability. These findings support accounts of visual expertise that emphasize attentional mechanisms and the role of active visual exploration in learning.

## 1. Introduction

Expert performance is characterized by high levels of accuracy achieved with greater speed and efficiency than typical individuals [1]. Accounts of expert performance emphasize different aspects of perceptual and cognitive processes, with varying emphasis on how experts sample, encode and retain information in memory. A key distinction is in the degree to which different domains of expertise rely on ‘front-end’ attention mechanisms that focus visual information from the environment onto the retina and the ‘back-end’ perceptual processing and memory storage [2,3].

There has been growing interest in a naturally occurring form of face recognition expertise found in individuals known as ‘super-recognizers’. Super-recognizers sit at the extreme high end of a face-recognition ability spectrum that varies continuously across the broader population [4–6]. Evidence suggests that face recognition ability is a cognitive trait: it has a strong genetic basis [7], dissociates from other intelligence and object recognition abilities [8,9; cf. 10,11], is resistant to training [12–14] and is stable over time [15,16]. Given the key role of face identity processing in social behaviour of primate species, and the dedicated neural systems that have evolved for this task [17], understanding the perceptual and cognitive processes underpinning super-recognizers’ abilities provides an important window into the nature of biological visual expertise.

One way to examine the cognitive and perceptual differences that underpin face identity processing ability is to ask whether the use of facial information differs in high and low performers. For example, one study tested the impact of replacing different facial features on identity matching in super-recognizers and typical viewers, and found similar sensitivity in these groups to feature-replacement conditions [18]. However, different approaches have produced contradictory results. For example, Davies-Thompson *et al.* [19] reported enhanced detection of changes in the nose region in super-recognizers relative to typical viewers, whereas other studies have linked superior recognition performance to increased reliance on the eye region [20–22]. One reason for these contradictory findings could be that the facial features that are most diagnostic of face identity—such as the eyes or nose—vary across different face identity decisions [12].

In contrast, eye-tracking studies have found consistent differences in the way that super-recognizers actively sample face information. For example, they tend to fixate on faces more often than typical viewers when viewing social scenes [23,24]. Similarly, Dunn *et al.* [25] found that super-recognizers made more fixations, and distributed these fixations more broadly across the face when studying unfamiliar faces in the learning phase of a face recognition task [26]. Interestingly, these types of visual exploration behaviours have been associated with enhanced memory for a variety of visual stimuli [3,27,28], suggesting that this may play a functional role in supporting face recognition abilities of super-recognizers.

**Figure 1.**
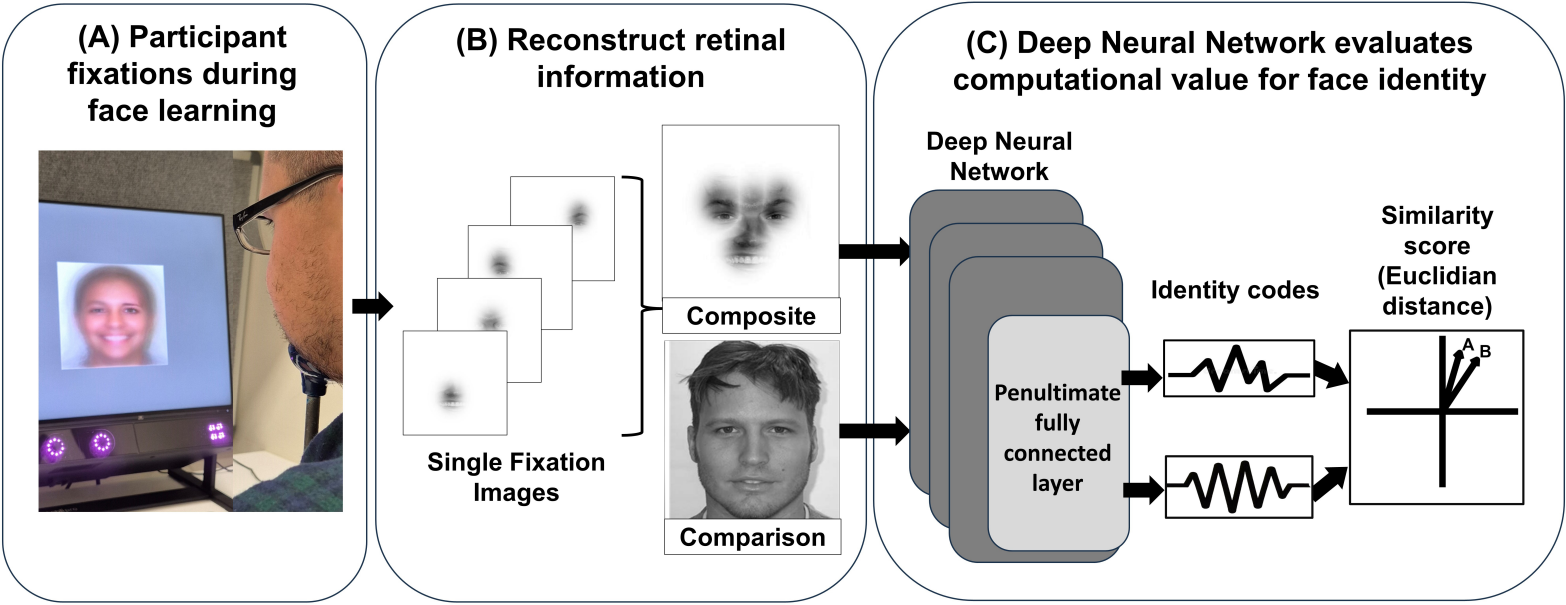
Quantifying the computational value of face identity information sampled by participants’ eye fixations. (A) Using fixation data from the learning phase of a gaze-contingent eye-tracking recognition memory experiment ([25]; see https://osf.io/xtjzh for an example trial), we (B) generated images of retinal information sampled with each fixation using retinal filter models [29,30]. These single fixations were combined into a composite image of retinal information for each face that a participant viewed when learning unfamiliar faces. (C) Retinal information images were then compared with a full, unaltered image of either the same or different identity (in this example, same) using DNNs to extract face-identity codes from the penultimate fully connected layer, and these were used to compute the face similarity score for the pairing. We quantified the amount of face identity information contained in the retinal information as the accuracy of these DNN similarity scores in discriminating matching from non-matching identity pairs. Further details are provided in §4.

While there is accumulating evidence of differences in super-recognizers’ visual exploration behaviour, it remains uncertain whether this leads to more robust representations of face identity. To address this question, we develop a novel method for quantifying the information content of eye movement-based visual samples made while participants learn new stimuli. As shown in figure 1, we used eye-tracking data from a previous study by Dunn *et al.* [25] to reconstruct the retinal information that had been actively sampled by participants as they learnt unfamiliar faces. In this study, participants viewed full-face images and also subsamples of face information through a gaze-controlled aperture centred on the viewer’s fixation. This technique allows participants to engage in natural, active visual exploration, while also enabling precise control over visual information available at each fixation—making the dataset uniquely suited to investigating the contribution of visual exploration behaviour to face recognition ability. Samples of retinal information were reconstructed from participant fixation data at each spotlight aperture size and assessed for their computational value using deep neural networks (DNNs) trained for face identity recognition.

Facial recognition algorithms have been used to quantify identity information in face images in prior work [31]. However, DNNs have produced large gains in the accuracy of facial recognition technology that substantially exceed what was achievable based on pre-DNN technology [32] and they now achieve very high accuracy that is at least equivalent to the very best human performers [33,34]. Therefore, DNNs appear to provide a near-optimal model for computing the face identity information available in images. We used them here to test whether the superior face recognition abilities in super-recognizers are predicated on face information encoded at the level of the retina.

## 2. Results

### (a) Comparing the computational value of face information sampled by typical viewers and super-recognizers

**Figure 2.**
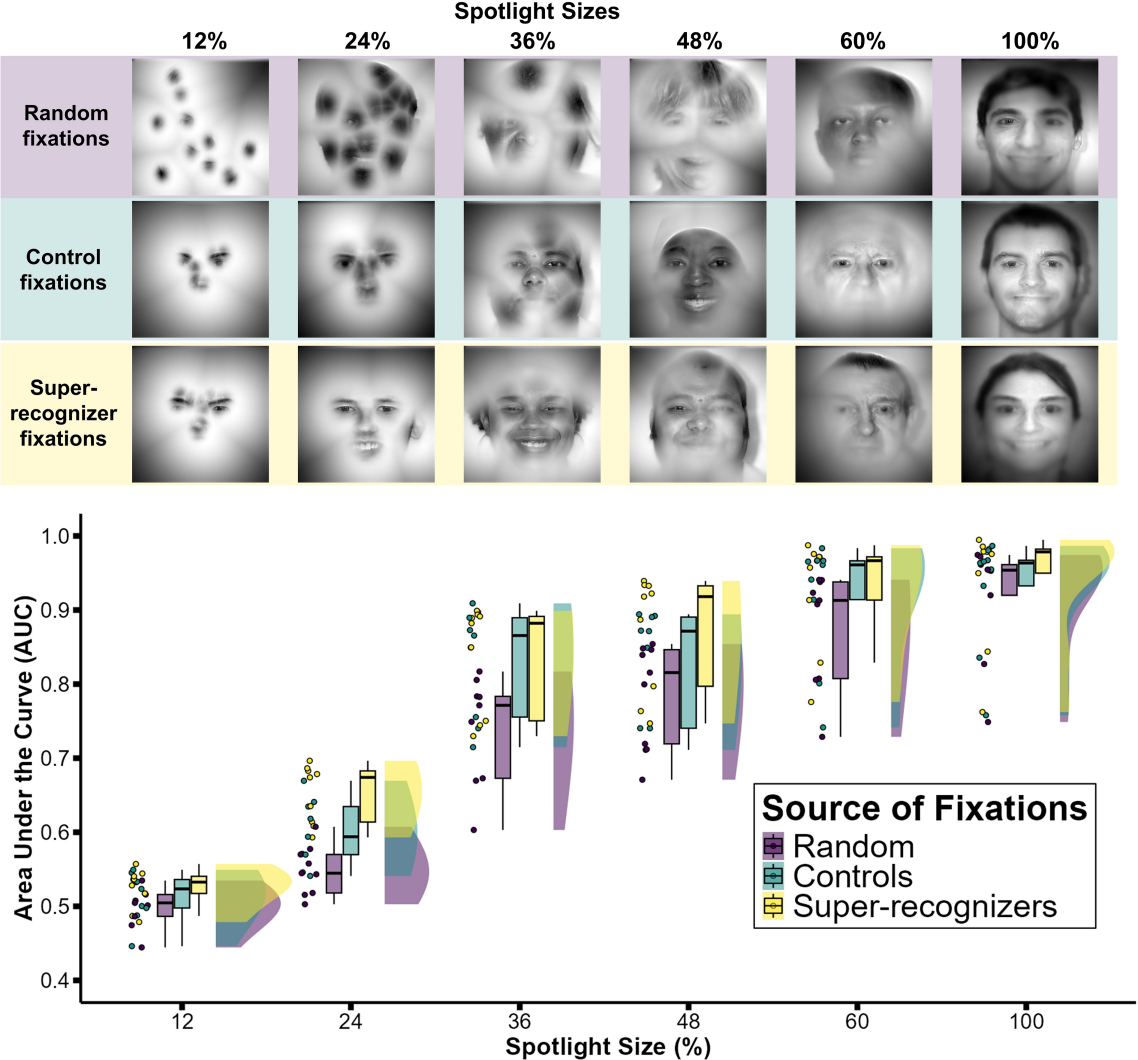
Super-recognizers sample computationally higher values of retinal information during a face learning task. Example composite images for each spotlight size and each of the information sampling types are shown in the top panel. The bottom panel shows identity matching accuracy (AUC scores) for nine DNNs. Across six spotlight sizes, matching accuracy was higher when using information sampled by super-recognizers than by typical viewers or random generation.

We tested whether the computational value of face information sampling differed between typical viewers and controls, and also whether information sampled by these groups exceeded random samples of information within the face. To quantify face identity information in retinal information (figure 1B), we calculated the extent to which DNN-derived similarity scores discriminated between matching and non-matching identity pairs (figure 1C), using area under the receiver operator characteristic curve (AUC). Figure 2 shows AUC scores for each of the gaze-contingent aperture viewing conditions and for each of the information sampling types (random sampling, control viewers and super-recognizer viewers).

Data shown in figure 2 were analysed using a generalized linear mixed model (GLMM with logit link function, GAMLj, package in Jamovi v. 2.3 [35]). The fixed effects were the size of the spotlight with which participants viewed the faces (12%, 24%, 36%, 48%, 60% and 100%; figure 1), the source of sampling fixations (random, super-recognizers, controls) and their interaction.^1^ In the mixed-effects model, DNNs were included as random intercepts to account for variability across networks. The dependent variable was the AUC score for each combination of spotlight size and source of fixation across all DNNs (i.e. for each DNN, we had 18 AUC scores). Comparing potential models, we found that the logistic model (Bayesian Information Criterion (BIC) = −416.73) provides a better fit than a linear model (BIC = −207.16) or quadratic model (BIC = −407). The logistic model also better accounts for the bounded AUC values.

Visual inspection of figure 1 shows numerically higher accuracy for super-recognizers than controls and random fixations at each spotlight size. In support of this, we found that while the main effects of information sampling type ($\chi_{1}^{2}$ = 36.64, *p* < 0.001) and spotlight size ($\chi_{1}^{2}$ = 27 816.68, *p* < 0.001) were significant, the interaction term was non-significant (*$\chi_{2}^{2}$* = 5.94, *p* = 0.051). These main effects showed that the DNNs’ performance improved as spotlight size increased (exp(*b*) = 2.58, CI = [2.55, 2.61]); when using information sampled by controls compared with random fixations (exp(*b*) = 0.74, CI = [0.63, 0.86], *z* = 3.77, one-tailed *p* < 0.001); and when using information sampled by super-recognizers compared with controls (exp(*b*) = 1.21, CI = [1, 1.48], *z* = 1.91, one-tailed *p* < 0.05). Together, these findings show that face information sampled by humans has higher computational value than randomly sampled information. Moreover, super-recognizers sample information with higher computational value relative to control participants.

### (b) Is the superior ability of super-recognizers owing to the greater quantity of face information they sample?

Super-recognizers’ gaze patterns are more broadly distributed across the face than typical viewers [25], meaning that they encode wider regions of the face. Because of this, we tested whether the greater computational value of super-recognizer retinal sampling could be explained by the greater volume of information alone, or whether they also sample higher*-quality* information.

To control for the amount of face information available across fixation sources, as well as potential differential difficulty in face matching across trials, we computed the proportion of total information from the original faces that was preserved in the reconstructed images. The proportion of total information was calculated according to Papinutto *et al*.’s [29] approach, based on the Structural SIMilarity index [36] and the pixel test [37].

Unlike the previous analysis, which used AUC as a summary measure of accuracy across trials, this analysis required a trial-level dependent variable to account for variability in individual face comparisons. We therefore used similarity score correctness as the dependent variable, which reflects whether the DNNs correctly classified match/non-match trials. For match trials, higher similarity scores indicated correct classification; for non-match trials, the scores were reversed so that lower similarity indicated correct rejection. This trial-level approach allowed us to assess how fixation source, spotlight size and total information influenced DNN accuracy at the trial-level, providing a more precise test of whether super-recognizers sample more diagnostic facial information. Similarity score correctness was analysed using a GLMM with source of sampling fixations, spotlight size and proportion of total information as fixed effects, and DNNs and image trials as random effects.^2^ The logit link function accounts for the fact that the dependent variable—similarity score correctness—is bounded and offers the smallest BIC compared with a linear model (BIC GLMM = −481 563.28).

Unsurprisingly, the DNNs’ performance at the trial level increased as spotlight size and the total amount of information available increased ($\chi_{1}^{2}$ = 233.81, *p* < 0.001 and $\chi_{1}^{2}$ = 131.60, *p* < 0.001, respectively). More importantly, the main effect of fixation source was also significant ($\chi_{2}^{2}$ = 79.22, *p* < 0.001). The DNNs provided more accurate scores when given information sampled by controls compared with random fixations (exp(*b*) = 0.96, CI = [0.94, 0.98], *z* = 4.55, *p* < 0.001). Critically, DNNs’ accuracy was higher based on super-recognizers’ information compared with controls’ (exp(*b*) = 1.04, CI = [1.01, 1.06], *z* = 3.22, *p* < 0.001). Because we included total amount of information as a fixed effect in this model, this effect is independent of the quantity of information revealed in each face. Thus, in addition to engaging in more exploratory viewing behaviour, super-recognizers also selectively target regions of the face with higher diagnostic value than controls do.

## 3. Discussion

Our study shows that active visual exploration behaviour when learning new faces is functional for the task of face recognition. Typical viewers’ fixations captured face information of substantially higher computational value for identity processing when compared with randomly dispersed fixations—confirming viewing behaviour in average observers that is sensitive to stable sources of identity information in the face. Moreover, super-recognizers’ viewing behaviour captured information of higher computational value compared with typical viewers. These findings suggest that the perceptual foundations of individual differences in face recognition ability may originate at the earliest stages of visual processing—at the level of retinal encoding.

We found that super-recognizers sampled higher-value face information, even when controlling for information quantity. Thus, super-recognizers’ exceptional face recognition skills are not merely owing to gathering more information but can also be attributed to their capacity to selectively sample facial features with high computational value. The main effect of spotlight size and lack of interaction suggests that viewing behaviour differences in super-recognizers were functional across a range of viewing conditions—both when faces are partially occluded from the viewer, and when in full view.

Our finding that super-recognizers appear to target attention on regions with higher diagnostic value is partly consistent with the information-reduction hypothesis [38], which states that expertise allows people to learn to focus on task-relevant information and ignore task-irrelevant information. A recent review of gaze behaviours in different forms of expertise highlights that experts across several domains, including art, the military and geography, show greater attention through the number of fixations or dwell time on critical features, supporting the conclusion that they are better able to attend to more valuable information [2,39]. However, research in face recognition has defined ‘critical’ facial features—such as the eyes—as features that are more diagnostic for recognition than other features *on average* [7–9]. This approach is inconsistent with evidence that the features that are diagnostic for identity for one face are not necessarily diagnostic for another [12], implying that featural sources of identity information are specific to individual faces [40].

The complex nature of diagnostic features for face recognition may explain why super-recognizers distribute their gaze more broadly across the face, rather than focusing on single critical features. This pattern suggests that their enhanced performance is not owing to reduced information encoding, but rather to more extensive visual exploration during learning, which may support the discovery of critical features on a face-by-face basis. Our findings show that this active exploration is functionally beneficial for face recognition and is more pronounced in individuals with exceptional face recognition abilities. Future research should therefore aim to understand this process of active sampling in greater detail. For example, while our study focused on still images, it is also important to investigate how super-recognizers form robust memory representations of faces across successive fixations in more dynamic visual contexts, such as video.

Exploratory viewing behaviour is also found to be associated with higher memory ability across a range of image domains [3,27,28], but previous research has lacked objective methods for quantifying the value of specific features used by experts [2]. Given that neural networks surpass human classification performance across a wide range of visual object categories, the paradigm we have introduced in this study may offer valuable insights into the functional role of visual sampling and exploration in visual task performance more broadly.

Visual exploration appears to be a key facet of human visual expertise. Notably, current deep learning approaches used to model face recognition ability do not have front-end components that actively sample information processing [41]. Recent attention-based models for face recognition have demonstrated the value of integrating front-end components [42], particularly under challenging conditions such as occlusion [43] and by incorporating temporal dynamics through sequential sampling of visual input [44]. This parallels the importance of active visual exploration in human face perception, and so similar models that are trained from human-sampled data could help in developing biologically plausible models of the face processing system in humans.

In conclusion, our results indicate that individual differences in face recognition ability are, at least in part, driven by differences in the way that individuals encode face information on the retina. This places important constraints on theories of these individual differences. Much of the prior work in this field has focused on testing representational accounts that emphasize high-level visual processing mechanisms [45]; however, the results presented here imply that any representational differences are preceded by differences in the input information. Future work should aim to understand the development of these attentional mechanisms, their neural substrates, and how they support the visual system to form robust representations of identity.

## 4. Method

### (a) Source data on viewing behaviour during face learning

The viewing behaviour data from 37 super-recognizers and 68 typical viewers were sourced from Dunn *et al.* [25]. Super-recognizers in that study were recruited based on having a mean *Z* score above 1.7 across three face recognition tests (Glasgow Face Matching Test: [GFMT 46]; UNSW Face Test: [15]; and Cambridge Face Memory Test Long form: [CFMT+: 5]) and typical viewers were recruited from the general population. This selection criterion is more stringent than those typically used in the field [47,48]. Requiring consistently high performance across multiple tests helps ensure that results are not influenced by chance or temporary factors.

Dunn *et al.* [25] employed a gaze-contingent paradigm in which participants viewed faces through ‘spotlight’ apertures that restricted visual information to a region surrounding their point of fixation. The aperture sizes varied across trials, revealing 12%, 24%, 36%, 48%, 60% or 100% of the face at each fixation.

The experiment consisted of two phases: learning and recognition. In the learning phase, participants viewed individual face images for 5 s each. These faces were presented at random locations on the computer screen to prevent anticipatory fixations. In the recognition phase, participants were shown faces one at a time and asked to indicate whether each face was ‘old’ (previously seen) or ‘new’ (not seen before). Faces remained on screen until a response was made. Importantly, learning and recognition phases were blocked, with the spotlight aperture size kept consistent between the learning and recognition phases for each block. The order of spotlight size blocks was randomized across participants to control for order effects.

Face stimuli were selected from the Lifespan Database of Adult Facial Stimuli [49], which includes a diverse set of faces varying in gender, age and ethnicity. For each identity, two photographs were used: one with a neutral expression and one with a happy expression. These images were taken minutes apart to ensure consistency in appearance aside from facial expression.

Saccades and fixations in this study were defined based on angular speed. All data points with an angular speed >30° s^−1^ were categorized as belonging to saccades, whereas all the other data points were categorized as belonging to fixations. Moreover, (a) fixations that were within 0.5° of visual angle and 75 ms were merged, (b) fixations shorter than 50 ms were removed and (c) fixations longer than 3 s.d. from the mean fixation duration of each participant were removed.

### (b) Recreating retinal information using static images

To assess the computational value of human fixation data from Dunn *et al.* [25], we generated static, trial-level representations of the visual information sampled by participants. These representations were designed to be compatible with DNN analysis and to simulate the constraints of the early visual system, particularly the decline in acuity with increasing distance from the point of fixation.

For each trial, we applied a retinal filter [30] to the gaze maps, which reduced image resolution as a function of distance from the nearest fixation point. This approach mimics the foveated nature of human vision and follows similar methods used by Papinutto *et al* [29]. The filter parameters included viewing distance, stimulus size in pixels and a lossy parameter (Δ = 25). This process resulted in 11 054 static, foveated images across all participants.

To evaluate whether human fixation patterns provided more diagnostic information than random sampling, we created a matched set of control images. For each human-generated composite image, we generated a corresponding image using randomly sampled fixation locations. The number of random fixations was yoked to the number of human fixations per trial. To ensure comparability, we used a prototype face mask delineating key facial regions (eyes, nose, mouth, chin, cheeks, brow and forehead) and verified that the number of fixations falling within these regions was matched between human and random conditions.

Each composite image—whether based on human or random fixations—was created by combining individual fixation-based views into a single trial-level image. Examples of composite images are shown in figure 2. These images were then processed using a multi-task cascaded convolutional network [50] to detect and align faces. All images were resized to 224 pixels × 224 pixels for input into the DNNs.

### (c) Using deep neural networks to evaluate computational value for face identity

We analysed the accuracy of nine open-source DNNs to discriminate between the matching and non-matching identity image pairs described above. Our goal was not to compare the accuracy of these DNNs *per se*, but rather to ensure the generalizability of our approach across different neural network architectures and training protocols. Thus, the nine selected models span a range of architectures and training datasets commonly used in computer engineering facial recognition research (see electronic supplementary material, table S1, for details) and also in work that has aimed to evaluate DNNs as computational models of face processing in humans [41]. These include Visual Geometry Group (VGG) based models (e.g. VGG16 [51]), Residual Neural Network (ResNet) variants (ResNet34 [52], ResNet50 [52], Se-ResNet50 [53]) and FaceNet implementations [54]. Training datasets include VGGFace [51], VGGFace2 [55], MS-Celeb−1M [56], CASIA-WebFace [57] and FaceScrub [58], ensuring coverage of both large-scale and fine-tuned face recognition benchmarks. Libraries used for implementation include both Keras (https://keras.io/) and PyTorch [59], reflecting practical diversity in deployment.

For match trials, the DNNs compared a reconstructed image with an original image of the same identity, showing a different facial expression to ensure that the algorithms were performing identity and not image matching. For non-match trials, algorithms compared one reconstructed image with an original image of a different identity with a different facial expression. To create non-match pairs, each identity was paired with another identity with similar demographics (e.g. self-reported gender, ethnicity and age).

The final image dataset consisted of 22 108 match pairs and 22 108 nonmatch pairs such that each DNN generated 44 216 similarity scores. All images were resized to 224 pixels × 224 pixels for input into the DNNs. Similarity scores were based on the Euclidean distance between the feature vectors generated at the penultimate layer of each DNN. That is, in each trial, a feature vector from the reconstructed image was compared with the feature vector of the original comparison image. We inverted the normalized similarity scores across all DNNs, resulting in similarity scores ranging from 0 to 1, where higher scores were more indicative of a match and lower scores closer to 0 were more indicative of a non-match.

## Data Availability

Stimuli and data are available here: [60]. Supplementary material is available online [61].

## Footnotes

^1^
AUC ~ 1 + Information Source + Spotlight Size + Information Source:Spotlight size + (1 | Algorithm).

^2^
Answer Correctness ~ 1 + Information Source + Spotlight Size + Total Information Available + (1 | Algorithm) + (1 | Stimulus)

## References

[1] Ericsson KA, Lehmann AC. 1996 Expert and exceptional performance: evidence of maximal adaptation to task constraints. Annu. Rev. Psychol. **47**, 273–305. (10.1146/annurev.psych.47.1.273) 15012483

[2] Brams S, Ziv G, Levin O, Spitz J, Wagemans J, Williams AM, Helsen WF. 2019 The relationship between gaze behavior, expertise, and performance: a systematic review. Psychol. Bull. **145**, 980–1027. (10.1037/bul0000207) 31414844

[3] Voss JL, Bridge DJ, Cohen NJ, Walker JA. 2017 A closer look at the hippocampus and memory. Trends Cogn. Sci. **21**, 577–588. (10.1016/j.tics.2017.05.008) 28625353 PMC5659202

[4] Noyes E, Phillips PJ, O’Toole A. 2017 What is a super-recogniser? In Face processing: systems, disorders and cultural differences (eds M Bindemann, AM Megreya), pp. 173–201. New York, NY: Hauppauge.

[5] Russell R, Duchaine B, Nakayama K. 2009 Super-recognizers: people with extraordinary face recognition ability. Psychon. Bull. Rev. **16**, 252–257. (10.3758/pbr.16.2.252) 19293090 PMC3904192

[6] White D. 2025 What is special about super-recognisers? In From super-recognisers to the face blind: why are some people better at recognising faces? (ed. K Lander). Oxford, UK: Oxford University Press. (10.1093/9780191986802.001.0001)

[7] Wilmer JB, Germine L, Chabris CF, Chatterjee G, Williams M, Loken E, Nakayama K, Duchaine B. 2010 Human face recognition ability is specific and highly heritable. Proc. Natl Acad. Sci. USA **107**, 5238–5241. (10.1073/pnas.0913053107) 20176944 PMC2841913

[8] Richler JJ, Wilmer JB, Gauthier I. 2017 General object recognition is specific: evidence from novel and familiar objects. Cognition **166**, 42–55. (10.1016/j.cognition.2017.05.019) 28554084

[9] Shakeshaft NG, Plomin R. 2015 Genetic specificity of face recognition. Proc. Natl Acad. Sci. USA **112**, 12887–12892. (10.1073/pnas.1421881112) 26417086 PMC4611634

[10] Geskin J, Behrmann M. 2018 Congenital prosopagnosia without object agnosia? A literature review. Cogn. Neuropsychol. **35**, 4–54. (10.1080/02643294.2017.1392295) 29165034

[11] Walker DL, Palermo R, Callis Z, Gignac GE. 2023 The association between intelligence and face processing abilities: a conceptual and meta-analytic review. Intelligence **96**, 101718. (10.1016/j.intell.2022.101718)

[12] Dunn JD, Towler A, Popovic B, de Courcey A, Lee NY, Kemp RI, Miellet S, White D. 2024 Flexible use of facial features supports face identity processing. J. Exp. Psychol. Hum. Percept. Perform. **50**, 1143–1153. (10.1037/xhp0001242) 39347769

[13] Towler A, Kemp RI, Burton AM, Dunn JD, Wayne T, Moreton R, White D. 2019 Do professional facial image comparison training courses work? PLoS One **14**, e0211037. (10.1371/journal.pone.0211037) 30759105 PMC6373902

[14] Towler A, Kemp RI, White D. 2021 Can face identification ability be trained? Evidence for two routes to expertise. In Forensic face matching: research and practice (ed. M Bindemann), pp. 89–114. Oxford, UK: Oxford Academic. (10.1093/oso/9780198837749.003.0005)

[15] Dunn JD, Summersby S, Towler A, Davis JP, White D. 2020 UNSW face test: a screening tool for super-recognizers. PLoS One **15**, e0241747. (10.1371/journal.pone.0241747) 33196639 PMC7668578

[16] White D, Guilbert D, Varela VPL, Jenkins R, Burton AM. 2022 GFMT2: a psychometric measure of face matching ability. Behav. Res. Methods **54**, 252–260. (10.3758/s13428-021-01638-x) 34159512

[17] Deen B, Schwiedrzik CM, Sliwa J, Freiwald WA. 2023 Specialized networks for social cognition in the primate brain. Annu. Rev. Neurosci. **46**, 381–401. (10.1146/annurev-neuro-102522-121410) 37428602 PMC11115357

[18] Abudarham N, Bate S, Duchaine B, Yovel G. 2021 Developmental prosopagnosics and super recognizers rely on the same facial features used by individuals with normal face recognition abilities for face identification. Neuropsychologia **160**, 107963. (10.1016/j.neuropsychologia.2021.107963) 34284039

[19] Davies-Thompson J, Morgan D, Davis JP, Towler JR. 2024 Face feature change detection ability in developmental prosopagnosia and super-recognisers. Brain Sci. **14**, 561. (10.3390/brainsci14060561) 38928560 PMC11201608

[20] Royer J, Blais C, Charbonneau I, Déry K, Tardif J, Duchaine B, Gosselin F, Fiset D. 2018 Greater reliance on the eye region predicts better face recognition ability. Cognition **181**, 12–20. (10.1016/j.cognition.2018.08.004) 30103033

[21] Royer J, Blais C, Gosselin F, Duncan J, Fiset D. 2015 When less is more: impact of face processing ability on recognition of visually degraded faces. J. Exp. Psychol. **41**, 1179–1183. (10.1037/xhp0000095) 26168140

[22] Tardif J, Morin Duchesne X, Cohan S, Royer J, Blais C, Fiset D, Duchaine B, Gosselin F. 2019 Use of face information varies systematically from developmental prosopagnosics to super-recognizers. Psychol. Sci. **30**, 300–308. (10.1177/0956797618811338) 30452304

[23] Bobak AK, Parris BA, Gregory NJ, Bennetts RJ, Bate S. 2017 Eye-movement strategies in developmental prosopagnosia and ‘super’ face recognition. Q. J. Exp. Psychol. **70**, 201–217. (10.1080/17470218.2016.1161059) 26933872

[24] Linka M, Broda MD, Alsheimer T, de Haas B, Ramon M. 2022 Characteristic fixation biases in super-recognizers. J. Vis. **22**, 17. (10.1167/jov.22.8.17) PMC9344214 35900724

[25] Dunn JD, Varela VPL, Nicholls VI, Papinutto M, White D, Miellet S. 2022 Face-information sampling in super-recognizers. Psychol. Sci. **33**, 1615–1630. (10.1177/09567976221096320) 36044042

[26] Dunn JD, Miellet S, White D. 2024 Information sampling differences supporting superior face identity processing ability. Psychon. Bull. Rev. **32**, 801–811. (10.3758/s13423-024-02579-0) 39313677 PMC12000253

[27] Fehlmann B, Coynel D, Schicktanz N, Milnik A, Gschwind L, Hofmann P, Papassotiropoulos A, de Quervain DJ. 2020 Visual exploration at higher fixation frequency increases subsequent memory recall. Cereb. Cortex Commun. **1**, tgaa032. (10.1093/texcom/tgaa032) 34296105 PMC8153053

[28] Heisz JJ, Pottruff MM, Shore DI. 2013 Females scan more than males: a potential mechanism for sex differences in recognition memory. Psychol. Sci. **24**, 1157–1163. (10.1177/0956797612468281) 23696202

[29] Papinutto M, Lao J, Ramon M, Caldara R, Miellet S. 2017 The facespan—the perceptual span for face recognition. J. Vis. **17**, 16. (10.1167/17.5.16) 28549354

[30] Targino Da Costa ALN, Do MN. 2014 A retina-based perceptually lossless limit and a Gaussian foveation scheme with loss control. IEEE J. Sel. Top. Signal Process. **8**, 438–453. (10.1109/jstsp.2014.2315716)

[31] Rice A, Phillips PJ, Natu V, An X, O’Toole AJ. 2013 Unaware person recognition from the body when face identification fails. Psychol. Sci. **24**, 2235–2243. 10.1177/0956797613492986 24068115

[32] Phillips PJ. 2017 A cross benchmark assessment of a deep convolutional neural network for face recognition. In 2017 12th IEEE International Conference on Automatic Face & Gesture Recognition (FG 2017), Washington, DC, 30 May –3 June 2017, pp. 705–710. (10.1109/FG.2017.89)

[33] Phillips PJ *et al*. 2018 Face recognition accuracy of forensic examiners, superrecognizers, and face recognition algorithms. Proc. Natl Acad. Sci. USA **115**, 6171–6176. (10.1073/pnas.1721355115) 29844174 PMC6004481

[34] Towler A, Dunn JD, Castro Martínez S, Moreton R, Eklof F, Ruifrok A, Kemp RI, White D. 2023 Diverse types of expertise in facial recognition. Sci. Rep. **13**, 11396. (10.1038/s41598-023-28632-x) 37452069 PMC10349110

[35] The jamovi project. 2022 jamovi (v. 2.3). See https://www.jamovi.org.

[36] Wang Z, Bovik AC, Sheikh HR, Simoncelli EP. 2004 Image quality assessment: from error visibility to structural similarity. IEEE Trans. Image Process. **13**, 600–612. (10.1109/tip.2003.819861) 15376593

[37] Chauvin A, Worsley KJ, Schyns PG, Arguin M, Gosselin F. 2005 Accurate statistical tests for smooth classification images. J. Vis **5**, 659–667. (10.1167/5.9.1) 16356076

[38] Haider H, Frensch PA. 1999 Eye movement during skill acquisition: more evidence for the information-reduction hypothesis. J. Exp. Psychol. **25**, 172–190. (10.1037//0278-7393.25.1.172)

[39] Gegenfurtner A, Lehtinen E, Säljö R. 2011 Expertise differences in the comprehension of visualizations: a meta-analysis of eye-tracking research in professional domains. Educ. Psychol. Rev. **23**, 523–552. (10.1007/s10648-011-9174-7)

[40] Burton AM, Kramer RSS, Ritchie KL, Jenkins R. 2016 Identity from variation: representations of faces derived from multiple instances. Cogn. Sci. **40**, 202–223. (10.1111/cogs.12231) 25824013

[41] Phillips PJ, White D. 2025 The state of modelling face processing in humans with deep learning. Br. J. Psychol. (10.1111/bjop.12794) PMC13051013 40364689

[42] Liu W, Xu G, Wang E. 2025 Research on the Method of Face Recognition Based on Attention Mechanism. In Wireless sensor networks. CWSN 2024. Communications in computer and information science (eds L Sun, Y Chen), vol. 2341. Singapore: Springer. (10.1007/978-981-96-2186-6_15)

[43] Zhang M, Zhang Y, Zhang Q. 2023 Attention-mechanism-based models for unconstrained face recognition with mask occlusion. Electronics **12**, 3916. (10.3390/electronics12183916)

[44] He T, Jin X, S. X, Huang J, Chen Z, Hua X. 2021 Dense interaction learning for video-based person re-identification. arXiv 2103.09013. (10.48550/arXiv.2103.09013)

[45] White D, Burton AM. 2022 Individual differences and the multidimensional nature of face perception. Nat. Rev. Psychol. **1**, 287–300. (10.1038/s44159-022-00041-3)

[46] Burton AM, White D, McNeill A. 2010 The Glasgow Face Matching Test. Behav. Res. Methods **42**, 286–291. (10.3758/brm.42.1.286) 20160307

[47] Bate S, Portch E, Mestry N. 2021 When two fields collide: Identifying ‘super-recognisers’ for neuropsychological and forensic face recognition research. Q. J. Exp. Psychol. **74**, 2154–2164. (10.1177/17470218211027695) PMC8531948 34110226

[48] Ramon M. 2021 Super-recognizers - a novel diagnostic framework, 70 cases, and guidelines for future work. Neuropsychologia **158**, 107809. (10.1016/j.neuropsychologia.2021.107809) 33662395

[49] Minear M, Park DC. 2004 A lifespan database of adult facial stimuli. Behav. Res. Methods Instruments Comput. **36**, 630–633. (10.3758/bf03206543) 15641408

[50] Zhang KP, Zhang ZP, Li ZF, Qiao Y. 2016 Joint face detection and alignment using multitask cascaded convolutional networks. IEEE Signal Process. Lett. **23**, 1499–1503. (10.1109/lsp.2016.2603342)

[51] Simonyan K, Zisserman A. 2014 Very deep convolutional networks for large-scale image recognition arXiv preprint arXiv.

[52] He K, Zhang X, Ren S, Sun J. 2016 Deep Residual Learning for Image Recognition. In 2016 IEEE Conference on Computer Vision and Pattern Recognition (CVPR), Las Vegas, pp. 770–778. NV, USA. (10.1109/CVPR.2016.90)

[53] Hu J, Shen L, Sun G. 2018 Squeeze-and-excitation networks. In In Proceedings of the IEEE conference on computer vision and pattern recognition, pp. 7132–7141. (10.48550/arXiv.1709.01507)

[54] Schroff F, Kalenichenko D, Philbin J. 2015 FaceNet: A Unified Embedding for Face Recognition and Clustering. In 2015 Ieee Conference on Computer Vision and Pattern Recognition (Cvpr), pp. 815–823. (10.48550/arXiv.1503.03832)

[55] Cao Q, Shen L, Xie W, Parkhi OM, Zisserman A. 2018 VGGFace2: A Dataset for Recognising Faces across Pose and Age. In 2018 13th IEEE International Conference on Automatic Face & Gesture Recognition, pp. 67–74. 10.1109/fg.2018.00020.

[56] Guo Y, Zhang L, Hu Y, He X, Gao J. 2016 Ms-celeb-1m: A dataset and benchmark for large-scale face recognition. In In European conference on computer vision, pp. 87–102. Cham: Springer International Publishing. (10.48550/arXiv.1607.08221)

[57] Yi D, Liao S, Li SZ. 2014 Learning Face Representation from Scratch. arXiv 1–9. (10.48550/arXiv.1411.7923)

[58] Ng HWei. 2015 A data-driven approach to cleaning large face datasets. In 2014 IEEE International Conference on Image Processing, ICIP, pp. 343–347. (10.1109/ICIP.2014.7025068)

[59] Paszke A, Gross S, Massa F, Lerer A, Bradbury J, Chanan G, Chintala S. 2019 Pytorch: An imperative style, high-performance deep learning library. arXiv 1–12. (10.48550/arXiv.1912.01703)

[60] Dunn JD, de Lima Varela VP, Popovic B, Summersby S, White D, Miellet S. 2025 Super-recognisers sample visual information of superior computational value for facial recognition https://osf.io/tpxs5/10.1098/rspb.2025.200541187921

[61] Dunn JD, Varela V, Popovic B, Summersby S, Miellet S, White D. 2025 Supplementary material from: Super-recognisers sample visual information of superior computational value for facial recognition. Figshare. (10.6084/m9.figshare.c.8112194)41187921

